# An innovative microplastic extraction technique: The switchable calcium chloride density separation column tested for biodegradable polymers, polyethylene, and polyamide

**DOI:** 10.1016/j.mex.2024.102560

**Published:** 2024-01-07

**Authors:** Darya Rodina, Christian Roth, Wendel Wohlleben, Patrizia Pfohl

**Affiliations:** a Department of Chemistry, University of Rochester, Rochester, NY 14627, United States; b BASF SE, Carl-Bosch-Str. 38, 67056 Ludwigshafen, Germany

**Keywords:** *Switchable calcium chloride density separation column*, Microplastics, Extraction, Density separation, Environment, Complex matrices, Compost

## Abstract

Extracting microplastics from complex matrices poses challenges due to the potential impact of harsh chemical treatments on microplastic properties. For fate and hazard assessment reliable techniques are needed to not only quantify the particle number but also to assess the physicochemical properties of environmental microplastics with minimum changes induced by extraction. Here we present the method development for an innovative and non-destructive extraction protocol based on a switchable calcium chloride density separation column. In contrast to commonly reported extraction protocols, the presented technique is suitable for targeted microplastic property analysis (e.g., surface chemistry and texture) by keeping chemical treatments (such as oxidation and enzymatic digestion) to a minimum. By adjusting the temperature we can control the aggregate state of the highly concentrated salt solution, allowing to separate the microplastics from matrix by cutting of purified, solidified samples. Harsh chemical treatments are avoided, as well as obstruction of microplastic extraction by adsorption to matrix components when passing the tap at the bottom of traditional density separation funnels.

The use of microplastics that were prelabeled with a fluorescence dye helped to solve difficulties observed during method development by visual inspection before measurement of extraction efficiency: We spiked a blank compost with low-density polyethylene (LDPE) and polyamide (PA). Additionally, UV aged LDPE was used to demonstrate applicability to more hydrophilic, more environmentally relevant microplastics. The obtained initial results show high recovery of both unaged and aged LDPE over 97 wt.-% and an efficient compost removal but a lower and less robust recovery (between 68 and 18 wt.-%) for PA particles that are more challenging to extract due to an unfortunate synergistic combination of smaller particle size and higher density. Method adaptation to other microplastic types may still be necessary. In short:

• A low-cost and simple approach without oxidation to extract (pre-aged) microplastics from compost
• Method development by visual observation using fluorescent labelled microplastics and method validation by spike-recovery tests

## Graphical abstract

**Figure:**
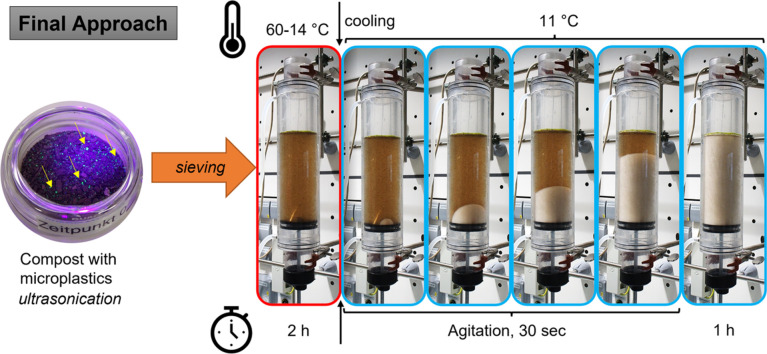

Specifications table

**Table:** 

Subject area:	Environmental Science
More specific subject area:	Microplastic extraction from complex environmental matrices
Name of your method:	Switchable calcium chloride density separation column
Name and reference of original method:	The extraction method using solid calcium chloride was newly developed and has not been mentioned in literature before. Calcium chloride salt solutions have been used for microplastic extractions, e.g. by Pfohl et al. [3]. and Crichton et al. [4]. and a column approach using a frozen column was introduced by Scopetani et al. [5].
Resource availability:	Saug- und Druckspritze 2014.1 - 1000 ml: https://oelluxx24.de/zubehoer/sonstiges-zubehoer/saug-und-druckspritze-2014.1-1000-ml?sPartner=oelfuxx_shopping&number=R12974&gclid=EAIaIQobChMI6JLU4pzk-AIVKYxoCR3d4gKeEAQYAyABEgK10PD_BwE Supplier: Pressol, article no. R12974, 5.4 × 6.5 × 9.5 mm Lauda RC6 mit RCS Thermostat - Gemini BV: https://www.geminibv.de/labware/lauda-rc6-mit-rcs-thermostat/ Supplier: Lauda, article no. 04733, temperature range: −35 °C to +150 °C Branson Digital Sonifier SFX 550 https://www.emerson.com/de-de/catalog/branson-sfx550-de-de Supplier: EMERSON, 13 mm Horn, 240 V CE, article no. 101–063–971R 1 mm wire mesh sieve https://www.retsch.com/products/sieving/test-sieves/mesh-sieves-other-sizes/ Supplier: Retsch, ø 100 mm, 1 mm mesh size, article no. 60.106.001000

## Method details

### Materials and reagents

For protocol development and visualization purposes, 80 g of a cryomilled (Retsch ZM 200 ultra centrifugal mill, <0.5 mm) and prelabeled biodegradable polyester blend (BASF SE, ρ = 1.25 g/cm^3^) was spiked into 1200 g compost (water content 49.4%) and dried at 58 °C until no further weight loss was observed [6]. As described before [6] the prelabeled blend was synthesized by compounding 3 kg of the biodegradable polyester with 3 g of the fluorescence dye Lumogen F Yellow 083 (BASF SE, 0.1% in weight) at 180 °C on a mini-extruder Rheomex CTW 100. The advantage of using microplastics labelled with a fluorescence dye is the possibility of visual inspection and photo capturing.

For method validation, we used a blank compost (compost without intentionally added microplastics; water content 49.4%, obtained by OWS Belgium), which was dried at 58 °C until no further weight loss was observed. For spike experiments we used cryomilled LDPE <250 µm (LyondellBasell), PA-6 <80 µm (BASF SE) and the same LDPE, which was UV pre-aged for 1000 h according to ISO 4892 (relative humidity 75%).

The 50 wt.-% (ρ = 1.5 g/cm^3^, pH = 6) calcium chloride (CaCl_2_) solution (anhydrous, >93.0 %, Sigma Aldrich) needs to be freshly prepared, and temperature should be kept around 60 °C to prevent spontaneous crystallization. We found handling and adjusting the aggregate state of anhydrous CaCl_2_ was more controllable than with a less purified technical CaCl_2_ (VWR Chemicals) or calcium chloride dihydrate (analytical grade, Bernd Kraft). In addition, better results were obtained using 50 wt.-% instead of 55 wt.-% CaCl_2_ solution, which was also tested in some of the reported approaches.

### Preparatory

Several approaches were tested during method development leading to different observations, challenges, and promising results. The method development itself was based on visual inspection using a fluorescent labelled biodegradable polyester blend (ρ = 1.26 g/cm^3^) in a first approach. Applying UV light we could easily observe how the particles were floating to the meniscus and where they were stuck (see below). These observations helped us to decide on the sedimentation time before starting the crystallization and where to cut the column to finally choose the experimental conditions for the final approach and the results were verified by gravimetric spike-recovery tests using LDPE and PA-6. Here we report four approaches and discuss their benefits and limitations. This method is predominantly based on density separation, since $\;\rho_{CaCl_{2}}$>$\;\rho_{polymer(tested)}$, leading to flotation of microplastics to the meniscus of the solution during extraction, while heavy compost components will sediment or will be dispersed within the column [7], [8], [9]. Microplastic types with a density higher than the chosen CaCl_2_ solution (ρ = 1.5 g/cm^3^) are not suitable for the presented extraction technique. Crystallization of the liquid CaCl_2_ can be activated by mechanical agitation in the cold (Video 1). The solid CaCl_2_ can afterwards be removed by pushing it out of the column. Using a saw, the upper 3–5 cm of the CaCl_2_ column containing the microplastics can be cut, the rest of the column containing compost components can be discarded. Efforts to recover calcium chloride containing the sediment for multiple purifications were impeded by occurrence of spontaneous crystallization and prevented us from recycling the material. Gentle heating (max. 60 °C) of the solidified top part of the CaCl_2_ column leads to liquefaction and the sample solution can be used in the next purification step. The purification steps can be repeated several times to remove the dispersed compost components in each step (washing steps: the compost solution is diluted in each step, while microplastics concentration will be maintained). Since oxidation and acidic/basic conditions are avoided, this approach is also suitable for e.g. biodegradable or partially degraded microplastics, that might be affected by harsh chemical treatments (e.g. changes in surface chemistry, texture and particle size distribution induced by the sample treatment) [1,2].

### Sample preparation

Subsamples of 1 g blank or spiked compost were taken and dispersed in 20 mL of ultrapure water using ultrasonication (Branson Digital Sonifier SFX 550, 5 min, 40%) in an ice bath to avoid overheating of the sample.

#### Approach 1

**Fig. 1:**
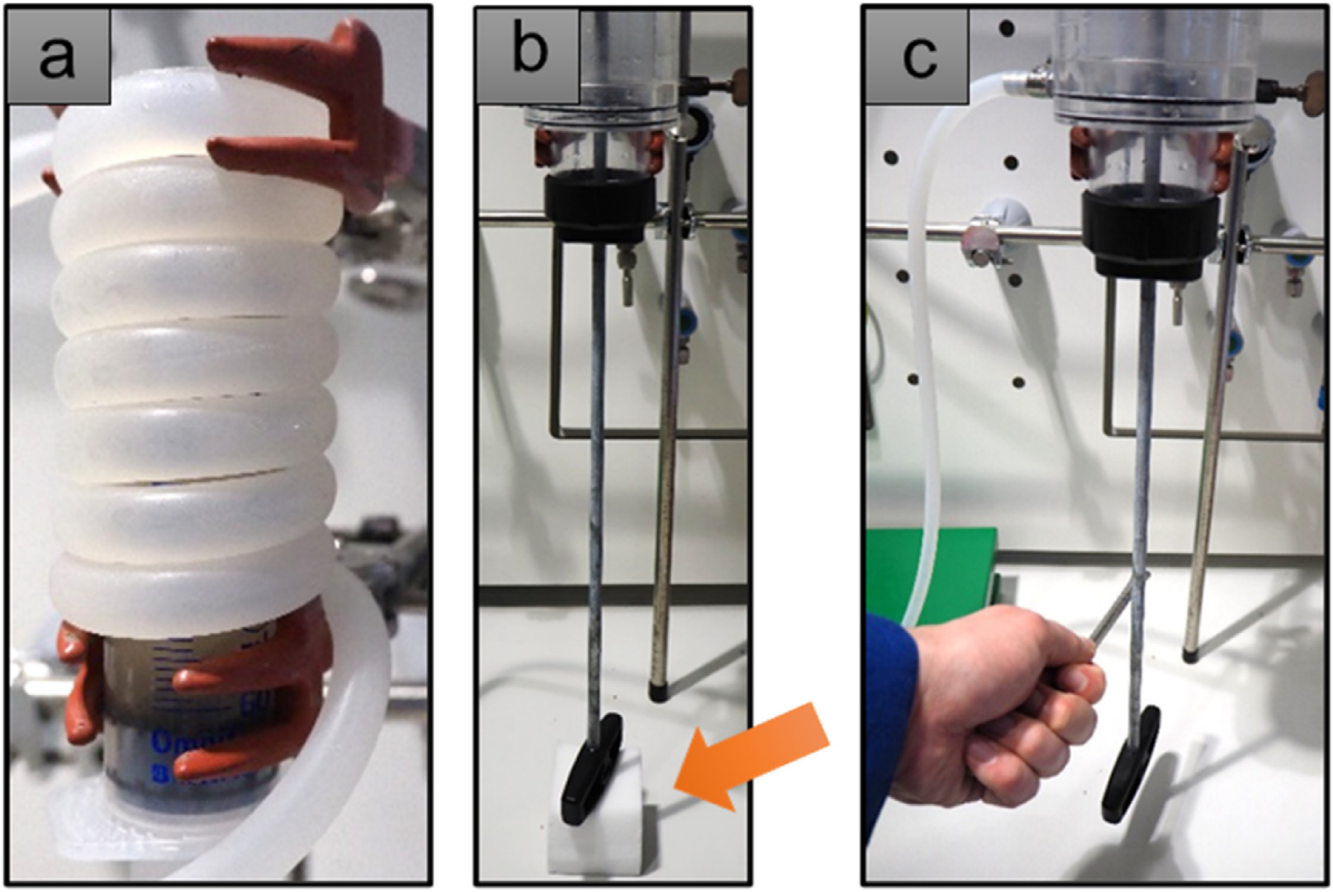
Method development for the switchable calcium chloride density separation. a) Tube set-up for cooling (approach 1); b) Foam material to prevent spontaneous crystallization induced by vibrations from the surroundings; c) Tapping of the calcium chloride solution in the cold state to induce crystallization.

300 mL of 55 wt.-% CaCl_2_ solution (40 °C) were pre-filled into the column (PRESSOL Saug- und Druckspritze, 1000 mL, upper part removed, vertically installed in the hood) and the dispersed sample was added, passing a 1 mm sieve. The sieve size was chosen to focus on smaller fragments but can be adapted according to the needs of future users. 50 mL of water were used to rinse the vial. A tube (continuous flow of water with *T* = 60 °C) was wrapped around the column for 2 h sedimentation time (Fig. 1a). Then the water temperature was cooled down to 5 °C (30 min). Crystallization could be started by mechanical agitation of the CaCl_2_ solution (tapping with a spatula against the push rod, Fig. 1c, video). The crystallization starts immediately without resuspension of denser particles in the sediment. After 2 h of crystallization in the cold the solid column was removed, but the core was still liquid, implying that the cooling mechanism needed to be improved.

#### Approach 2

**Fig. 2:**
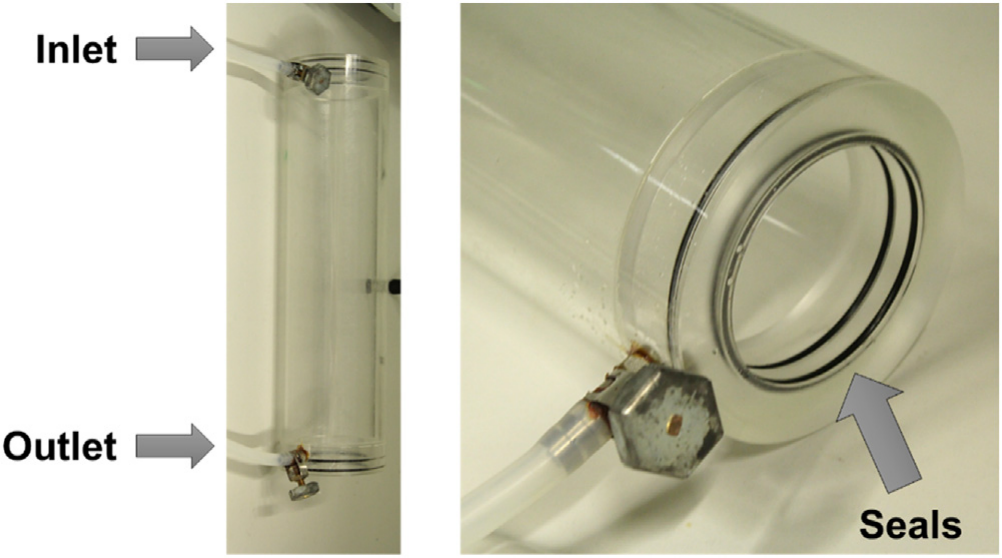
Housing fabricated at BASF SE. This part can be connected to the thermostat on two ends, enabling circulation of water through the system for heating and cooling.

**Fig. 3:**
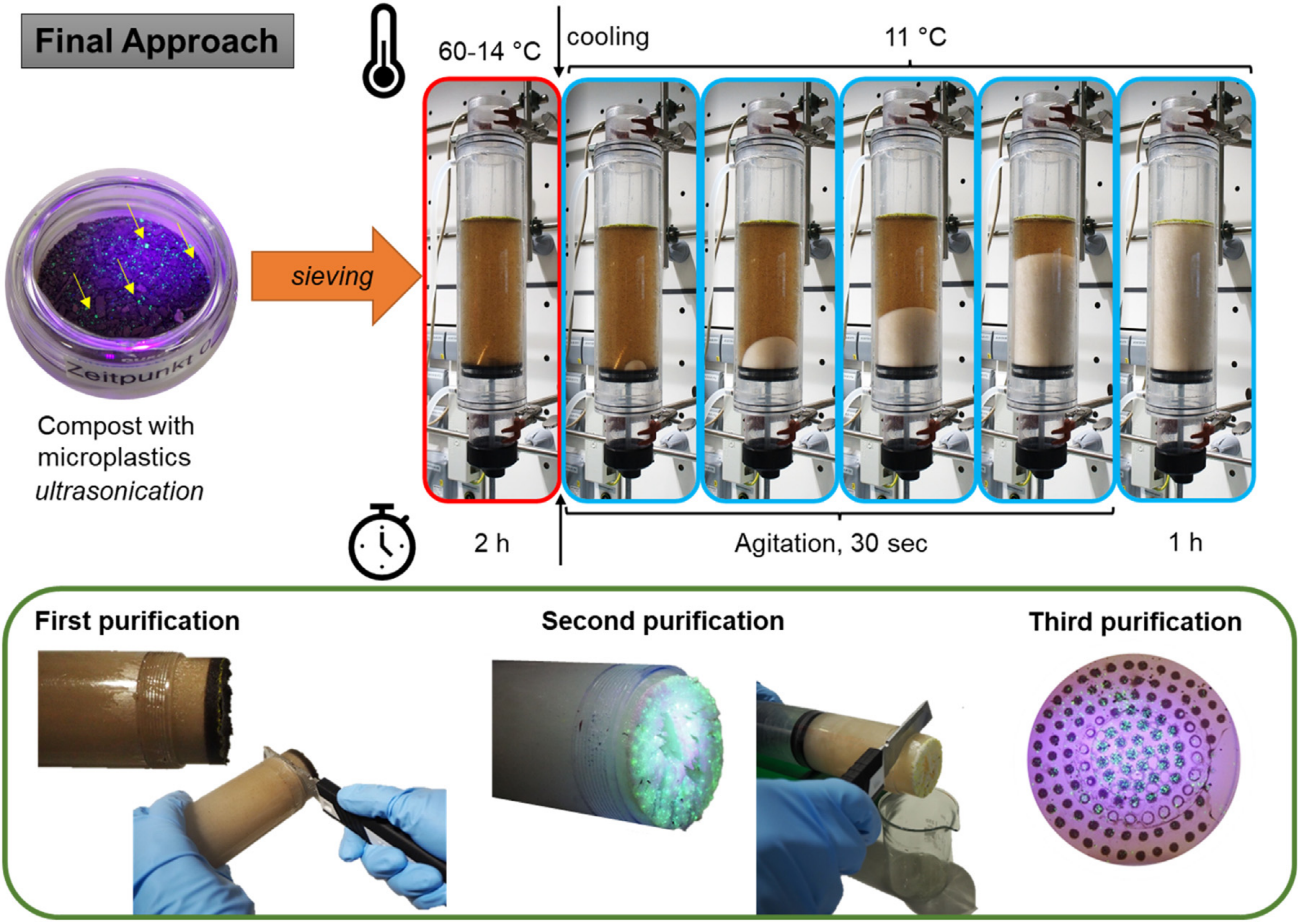
An innovative microplastic extraction protocol: the switchable calcium chloride density separation (final approach). The aggregate state of the calcium chloride solution can be adjusted with temperature and agitation.

In this approach 50 mL of 50 wt.-% CaCl_2_ were pre-filled into the column, the remaining solution (450 mL) was used to rinse the sample out of the vial after ultrasonication. The new set-up included a housing (Fig. 2) fabricated at BASF SE to increase the area for heating and cooling the column (compared to the tube set-up where only part of the water-containing tube was in contact with the outer column wall). The housing itself is a plastic column that fits exactly around the Pressol column. It can be connected to the thermostat on two ends, enabling the water to circulate through the system. The temperature is controlled by the connected thermostat. The housing is not double walled, but seals prevent the water from leaking out of the housing. In this way the water is directly in contact with the walls of the inner Pressol column, providing efficient cooling and heating of the system. With this set-up, the crystallization time could be reduced to 30 min and the CaCl_2_ column was completely crystalline, enabling the cutting of the upper layer (Fig. 3).

Additional observations: Several points are important to consider in further method development: 1) Vibrations from the surroundings can induce spontaneous crystallization when the temperature is low (here from the thermostat). This could be avoided by placing a foamed material underneath the push rod (Fig. 1b). 2) Air bubbles in the CaCl_2_ solution are formed next to the column walls, small particles can be trapped when getting in contact with those. This could be solved by adding a magnetic glass stir bar into the column: With the aid of an external strong magnet, one could move the stir bar along the column wall to remove the air bubbles. 3) During cooling from 60 °C to 5 °C the convective flow of heat transported prelabeled microplastics throughout the column. This means that additional sedimentation time in the cold is needed to allow the particles to float again (approach 3). Additionally, control experiments with and without a stir bar suggests that it aids crystallization process as it vibrates following agitation of the column.

#### Approach 3

In this approach the stability of the CaCl_2_ solution at lower temperature was tested. Using the foam material to prevent spontaneous crystallization (Fig. 1b), the 2 h sedimentation time at 60 °C could be avoided. Instead, the column was filled with warm CaCl_2_ (40 °C) at room temperature and the housing was afterwards directly flushed with cold water (12 °C). After 2 h of sedimentation in the cold, crystallization was activated, and the column was cooled for additional 60 min until it could be removed.

#### Final approach

The adjustments made in approaches 1–3 were used in the final approach (Fig. 3). The concentration of the CaCl_2_ solution was set to 50 wt.-%. With this approach, microplastic recovery tests were conducted. When compost was spiked with different polymers, the temperature of crystallization has slightly changed provoking spontaneous, unwanted crystallization. To obtain better control, the cooling protocol has been changed to slow cooling to 14 °C over 1 h 45 min, then, the mixture was cooled to 11 °C over 15 min and external stress was applied to initiate crystallization. The column was left for 60 min at 11 °C to assure sufficient solidification. Additionally, ten drops of surfactant (Lutensol TO7, BASF SE) 0.1% in water were added to each column before the addition of the CaCl_2_ solution. Finally, to achieve optimal compost removal, each sample was subjected to three rounds of purification: The upper 5 cm containing the microplastics were cut and liquified by slight heating (60 °C max). 50 mL of 50 wt.-% CaCl_2_ were placed in the column and 450 mL of 50 wt.-% CaCl_2_ were used to flush out the beaker with the liquified CaCl_2_ containing the microplastics. In this way the separation procedure could be repeated. This protocol was used for method validation and polymer recovery studies.

## Method validation

### Control experiment

Blank compost was used to run the purification in duplicates to calculate the matrix removal efficiency (Table 1). The average result of two runs demonstrated 97.6 % reduction in compost mass following three rounds of purification. This value was used to calculate polymer recovery when the compost samples were spiked with known microplastic mass.

**Table 1:** Results of blank experiments using compost samples with average matrix removal efficiency of 97.6%.

Experiment	Compost mass prior purification (g)	Compost mass post purification (g)	Percent matrix removal (%)	Range of duplicates (%)
1	1.1149	0.0344	96.9	0.91
*2*	*1.1915*	*0.0214*	*98.2*	

### Microplastic extraction

**Fig. 4:**
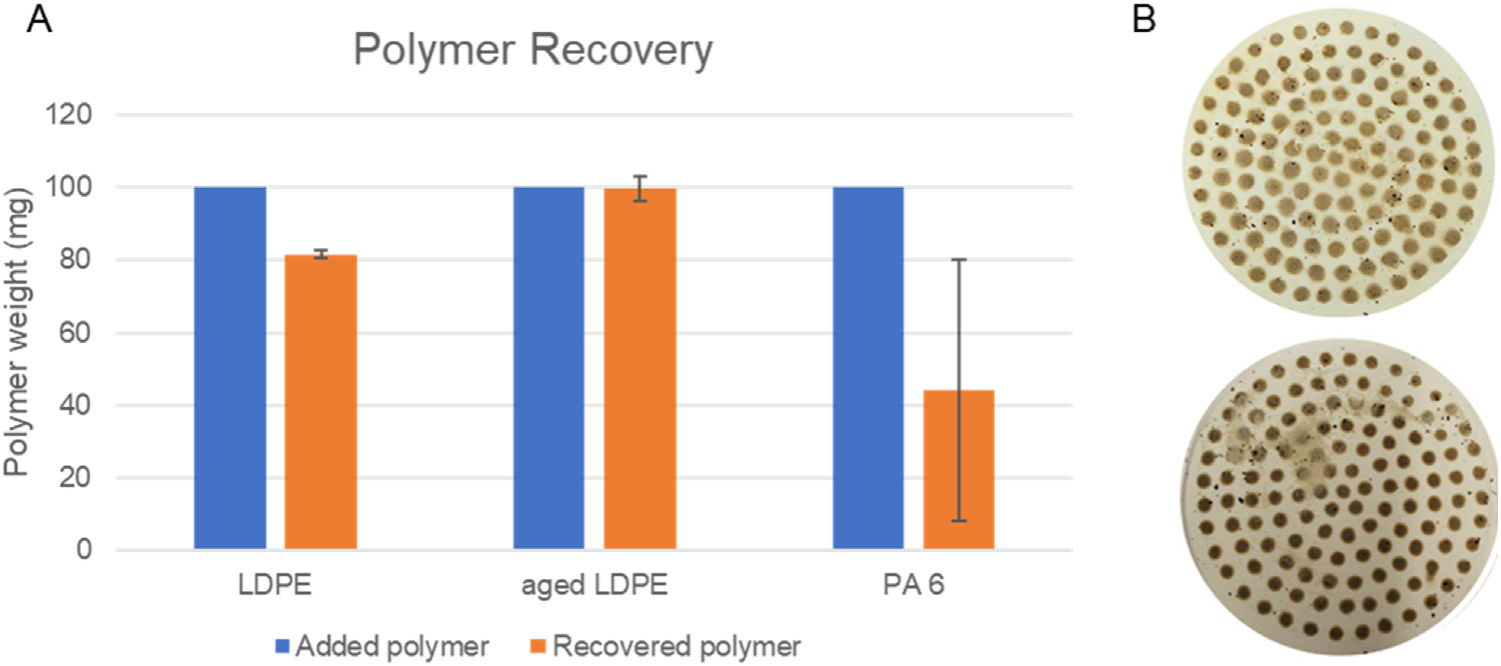
A. Polymer recovery results of LDPE, aged LDPE, and PA 6 in duplicates; B. Top filter photo after blank compost experiment, bottom photo of the filter with LDPE after the extraction.

The final approach was used to determine the microplastic recovery from compost samples. LDPE (109.1 and 144.9 mg) was added to the compost prior sonication and carried through the established extraction protocol. Two runs yielded 80.8 % and 82.4% LDPE mass recovery respectively (Fig. 4), which is comparable to recoveries obtained in the literature [10]. The polymer recovery results with UV aged LDPE are 101.9 % and 97.4 % respectively from 142.5 to 113.4 mg of polymer added prior extraction. This result highlights the method's advantage, as the aged polymer, typically vulnerable to chemically harsh extraction conditions (such as digestive treatments based on oxidation) [11,12], seemed to exhibit even higher recovery compared to the pristine material. Due to UV aging the hydrophilicity of LDPE is increased [13], which might reduce the undesired interaction with compost and CaCl_2_.

However, the recovery of 174.6 and 151.3 mg PA-6 microplastics was lower compared to LDPE and not consistent between the two runs with percent recovery of 69.6 and 18.6 %. One possible explanation is the higher density of PA-6 compared to LDPE, along with the significantly smaller particle sizes (compare SI Fig. 2 and Fig. 4 in our previous publication [1]). Smaller particles and increased density synergistically lead to a prolonged flotation time. In this case, controls are important to determine the extraction efficiency. Future research should investigate the minimum particle sizes that can be extracted with this method, as well as the influence of microplastic densities and adapt the method accordingly.

To sum up, the advantage of the presented extraction technique compared to commonly used techniques is, that chemical treatments are kept to a minimum by avoiding oxidation, organic solvents or enzymatic digestion. The reported techniques in literature are promising for microplastic quantification, even though reported microplastic recoveries also range from <10–100% [10,^14^,15]. However, investigating the physicochemical properties of environmental microplastics (such as surface chemistry and texture of aged microplastics, biofilm formation and more) is equally important and cannot reliably be assessed by using harsh chemical treatments during extraction [1,2]. In addition, using a column instead of a conventional separating funnel, the upper part of the solution containing microplastics does not need to pass through the lower outlet of the funnel, which might get obstructed [5,16]. Instead, it can be removed by pushing the solid column out at the upper end of the set-up. In this study we tested the applicability of the method to compost, but we assume that it could also be applicable to soils or sludge. For environmental matrices containing a high amount of inorganics, we would assume a higher matrix removal efficiency due to fast sedimentation of inorganics. In soil extraction, microplastics might be trapped in the sediment, but also this issue could be remediated by repeated treatment of the same sediment, as done in other protocols as well. The final approach considered all difficulties observed during method development (including negative results for the extraction of small PA particles) and we would like to encourage researchers to continue with the improvement and to perform recovery and polymer stability tests increasing the robustness of the presented method. For all studies that aim to investigate microplastics above 10 µm (Stoke's equation) [3], the present method is attractive due to its simplicity and the proven efficiency and purity of extraction. Only for smaller fragments, density separation longer than 2 h is typically required (depending on density) and can be attained with further method development.

## Ethics statements

Not relevant to this work.

## Funding

This research did not receive any specific grant from funding agencies in the public, commercial, or not-for-profit sectors.

## CRediT authorship contribution statement

**Darya Rodina:** Conceptualization, Methodology, Visualization, Writing – original draft, Writing – review & editing. **Christian Roth:** Conceptualization, Methodology, Investigation, Writing – review & editing. **Wendel Wohlleben:** Supervision, Conceptualization, Methodology, Project administration, Writing – review & editing. **Patrizia Pfohl:** Supervision, Conceptualization, Methodology, Visualization, Writing – original draft, Writing – review & editing.

## Declaration of Competing Interest

The authors declare the following financial interests/personal relationships which may be considered as potential competing interests:

Some of the authors are employees of BASF, a company producing and marketing polymers, including plastics.

## Data Availability

All data generated or analyzed during this study are included in this published article and its supplementary information files.

## Appendix Supplementary materials

Image, application 1
