# Supplementary material for: An innovative microplastic extraction technique: The switchable calcium chloride density separation column tested for biodegradable polymers, polyethylene, and polyamide
Source: MethodsX. 2024 Jan 7;12:102560. doi: 10.1016/j.mex.2024.102560 (PMC10825483; doi:10.1016/j.mex.2024.102560)
Supplement: Supplementary file 1 [file mmc1.docx]

Column Activation.mp4
